# Chromosomal instability and cancer: An insight into the rhythm of life

**DOI:** 10.4103/0971-6866.50861

**Published:** 2009

**Authors:** Babu Rao Vundinti, Kanjaksha Ghosh

**Affiliations:** National Institute of Immunohaematology (ICMR), K.E.M Hospital Campus, Parel, Mumbai, India

Kaur *et al,*[1] described chromosomal instability in breast cancer in this issue of the journal. The results are not surprising as many human cancers have chromosomal abnormalities and, frequently, specific abnormalities are associated with specific forms of cancer. These findings have emerged gradually over the past 35 years as increasingly better techniques have become available for the preparation and analysis of human chromosomes. Initial studies simply characterized gross chromosome abnormalities occurring in different types of cancer cells. More recently, molecular rearrangements that are associated with many chromosomal abnormalities have been characterized. Chromosomal instability refers to an increased rate of losing or gaining whole chromosomes or large parts of chromosomes during cell division. The consequence of chromosomal instability is an imbalance in the chromosome number and an increased rate of loss of heterozygosity (LOH). An increased rate of LOH is an important property of chromosomal instability because it accelerates the inactivation of the tumor suppressor genes.

It has long been considered that chromosomal instability, an end point of genomic instability, is an integral component of human cancer. The multiple phenotypes of genomic instability may induce various karyotypic abnormalities such as chromosomal translocations, deletions, inversions or duplications. Although chromosomal instability is defined as an increased rate of losing or gaining parts of chromosomes or whole chromosomes during cell division, CIN has only been formally demonstrated for whole chromosome losses. There is no assay at present that can reliably measure the rate of formation of changes at the subchromosomal level, such as deletions, inversions, rearrangements, amplifications, unequal sister chromatid exchange and gene conversion. Mechanisms leading to chromosomal instability have been suggested to involve the faulty DNA repair process, telomere loss of chromosomes and aberrant cell cycle control. Advancements in cytogenetic technology help in identifying submicroscopic chromosomal rearrangements in human cancers. The M-FISH or Spectral karyotyping colorized the cytogenetics and made it possible to identify marker chromosomes, also their origin, especially in solid tumors. Recently, the area of cytogenetics competed with molecular markers and was able to identify global losses or gains in the genome and showed its role in tumerogenesis.

Recently, the genes triggering chromosomal instability in humans and leading to cancers have been demonstrated. These genes include hBUB1, MAD2, BRCA1, BRCA2 and hcDC4.[2] The hBUB1 and MAD2 are required for the proper functioning of the spindle assembly checkpoint. This checkpoint modulates the timing of anaphase initiation in mitotic cells containing improperly aligned chromosomes and increases the probability of successful delivery of a correct chromosome set to each daughter cell. HcDC4 is an E3 ubiquitin ligase that is involved in regulating the G1–S cell cycle checkpoint by targeting proteins for destruction. Inactivation of hCDC4 leads to increased levels of cyclin E, the formation of micronuclei, defects in the execution of anaphase and chromosomal instability. BRCA1 and BRCA2 are involved in DNA repair and recombination, checkpoint control of the cell cycle and transcription.[3] Inherited mutations in BRCA1 and BRCA2 lead to high-grade familial breast cancer. Recently, the Fanconi anemia pathway was shown to be associated with BRCA1/BRCA2 proteins. The defects in these proteins were experimentally demonstrated to cause genomic instability.[4] The chromosomal instability syndromes such as Ataxia-Telangiectasia, Bloom syndrome, Nijmegen breakage syndrome, Cokayne syndrome, etc. have defects in the DNA repair pathway and are prone to cancer development. Hence, molecular studies are important in determining the genes involved in disease.

We have come a long way in understanding carcinogenesis. Starting with environmental carcinogens like polycyclic hydrocarbon somatic mutation, various kinds of clonal chromosomal abnormality activation of oncogenes, haploinsuffiency or deletion of tumor suppressor genes and involvement of deregulation of cell cycle pathway have all been described in the context of carcinogenesis or leukaemogenesis. Chromosomal instability is another component in this jigsaw puzzle and takes our understanding of this phenomenon a step further.
